# Editorial: The experimental models as a tool for studying therapeutic targets in COPD: lessons learned

**DOI:** 10.3389/fimmu.2026.1988969

**Published:** 2026-10-01

**Authors:** Fernanda Degobbi T Q S Lopes, Rodolfo P Vieira, Asghar Abbasi

**Affiliations:** 1 Laboratory of Experimental Therapeutics (LIM20), Department of Clinical Medicine, School of Medicine, University of Sao Paulo, Sao Paulo, Brazil; 2 Thoracic Surgery Research Laboratory (LIM61), Division of Thoracic Surgery, Instituto do Coracao do Hospital das Clinicas da Faculdade de Medicina da Universidade de Sao Paulo, Sao Paulo, Brazil; 3 Evangelical University of Goias (UniEvangelica), Laboratory of Pulmonary and Exercise Immunology (LABPEI), Anápolis, GO, Brazil; 4 Brazilian Institute of Teaching and Research in Pulmonary and Exercise Immunology (IBEPIPE), Department of Research and Development, Anápolis, GO, Brazil; 5 Respiratory Research Center, The Lundquist Institute for Biomedical Innovation at Harbor-UCLA Medical Center, Torrance, CA, United States; 6 Division of Respiratory Critical Care Physiology and Medicine, Harbor-UCLA Medical Center, Torrance, CA, United States; 7 College of Health Sciences, The University of Texas at El Paso, El Paso, TX, United States

**Keywords:** chronic obstructive pulmonary disease (COPD), experimental models, therapeutic targets, inflammation, translational research, precision medicine

Chronic obstructive pulmonary disease (COPD) is a heterogeneous lung disease characterized by persistent airflow limitation arising from complex pathological changes involving the airways and lung parenchyma. Its pathogenesis involves chronic pulmonary inflammation, oxidative stress, structural remodeling, and systemic inflammatory consequences that may contribute to extrapulmonary manifestations and comorbidities (1). Despite substantial advances in understanding these mechanisms, translating mechanistic discoveries into effective disease-modifying therapies remains challenging. Experimental models are therefore essential for dissecting COPD pathogenesis, establishing causal mechanisms, and evaluating potential therapeutic targets. However, no single experimental model reproduces the full pathological and clinical complexity of human COPD, and individual models differ considerably in the disease components they reproduce (2–4). Accordingly, model selection should be guided by the specific biological mechanism, disease phenotype, or therapeutic question under investigation.

The contributions to this Research Topic illustrate the complementary value of different experimental approaches. Nishino et al. demonstrated a protective role for hyaluronic acid synthase 2 (HAS2) against neutrophilic inflammation and emphysema using genetically modified mice and an elastase-induced model; specifically, Has2+/- mice displayed significantly increased neutrophil counts in bronchoalveolar lavage fluid and higher mean linear intercept values compared with wild-type mice, with increased G-CSF levels and attenuated TGF-β in the lungs. Moreira et al. showed in a chronic cigarette smoke (CS) model that IL-17 neutralization by IL-17- neutralizing antibody attenuated inflammatory, structural, and functional abnormalities; treatment was initiated in the fifth month of a six-month protocol, and the antibody reversed increases in mean linear intercept and restored tissue elastance and tissue damping, underscoring the therapeutic relevance of intervention timing in established disease. At another experimental level, Zhang et al. examined Drosophila melanogaster as a genetically tractable and high-throughput platform for investigating COPD-related mechanisms and identifying candidate therapeutic targets; the fly model shares conserved innate immune pathways, oxidative stress responses, and tracheal remodeling features with mammalian COPD, although the absence of adaptive immunity and alveolar structures limits direct extrapolation and requires validation in mammalian systems.

Several contributions demonstrate how experimental models are expanding therapeutic investigation beyond different inflammatory signaling pathways. Xie et al. synthesized the dynamic roles of macrophages, neutrophils, T lymphocytes, and other immune populations across experimental COPD models, and emphasized that immune responses and therapeutic effects may vary according to the inflammatory phenotype. Gong et al. combined human lung and serum samples, a CS-induced rat model, and bronchial epithelial cells to identify a protective role for FGF7 against epithelial injury, inflammation, and remodeling; AAV-mediated FGF7 overexpression reduced BALF levels of IL-1β, IL-6, TNF-α, TGF-β1, and ET-1 and activated the ADAM17/EGFR/ERK1/2, p38, and AKT signaling axes. Zeng et al. extended this mechanistic landscape to immunometabolism, highlighting how alterations in glucose, lipid, and amino acid metabolism may interact with chronic immune activation in COPD, identifying HIF-1α-driven glycolysis, fatty acid oxidation dysregulation, and PPAR-γ agonism as specific therapeutic axes. Similarly, Shi et al. integrated bioinformatics, machine learning, single-cell analysis, and experimental validation to identify six cellular energy metabolism-related biomarkers (CYP1B1, CA3, AHRR, MGAM, PNMT, and PLA2G1B; diagnostic AUC 0.814) and validated the regulatory role of PLA2G1B in epithelial cell inflammation, oxidative stress, and apoptosis, showing that PLA2G1B knockdown increased intracellular ROS and activated the apoptotic pathway in BEAS-2B cells. Huang et al. further explored pyroptosis and ferroptosis as potentially interconnected cell-death programs and proposed a framework in which smoke-induced Nrf2 epigenetic silencing drives ferroptotic lipid peroxide accumulation that triggers pyroptotic execution via caspase-11, positioning GPX4 as a molecular gatekeeper; the framework further appraises NLRP3 inhibitors, dupilumab, anti-alarmin biologics, and ferroptosis-directed agents within a biomarker-guided precision medicine strategy, while several proposed interactions still require direct experimental validation in COPD-specific systems.

Importantly, experimental discoveries ultimately need to be connected with human disease. Ma et al. provided such a translational perspective by demonstrating that circulating osteopontin was associated with disease severity and adverse outcomes in a prospective cohort of 281 acute exacerbation, 89 stable COPD, and 89 healthy control participants; serum osteopontin was positively associated with clinical severity scores and inversely correlated with pulmonary function, and independently predicted death and acute exacerbation risk in the first year, supporting its further investigation as a prognostic biomarker and as a potential link to underlying disease mechanisms. The need to extend experimental thinking beyond the lung is also emphasized by Abbasi et al., who examined muscle-lung crosstalk in experimental COPD and how systemic inflammation, mitochondrial dysfunction, altered protein metabolism, and candidate inter-organ mediators may contribute to skeletal muscle dysfunction and most importantly be modified by exercise training.

Together, these contributions provide several important lessons. First, there is no universal experimental model of COPD, rather, model selection should be guided by the specific phenotype, mechanism, or therapeutic question being investigated. Second, observations of altered biomarkers or pathways should, whenever possible, progress toward causal testing through genetic, pharmacological, or other targeted perturbations. Third, prevention and treatment paradigms should be clearly distinguished, because an intervention administered before or during disease development may not predict its effectiveness against established disease. Fourth, the heterogeneity of COPD requires increasing attention to immune and molecular phenotypes when interpreting experimental findings and therapeutic responses. Finally, COPD should be considered a multisystem disorder in which pulmonary, immune, metabolic, and extrapulmonary mechanisms interact.

Collectively, the articles in this Research Topic demonstrate that progress in COPD modeling may depend less on identifying a single “best” model than on strategically combining experimental systems according to their individual strengths and limitations. Integrating mechanistic discovery, causal experimental validation, phenotype-relevant models, and human translational evidence should strengthen therapeutic-target identification and ultimately facilitate the development of more precise interventions for COPD.
